# Ecological momentary assessments for patients with hereditary angioedema: a feasibility and acceptability controlled study

**DOI:** 10.3389/fdgth.2025.1693550

**Published:** 2026-01-12

**Authors:** Monica Parati, Luca Ranucci, Azzurra Cesoni Marcelli, Lorenza Chiara Zingale, Beatrice De Maria, Clara Gino, Aida Zulueta, Riccardo Sideri, Alessandra Gorini, Francesca Perego

**Affiliations:** 1 Department of Internal Medicine and Rehabilitation, Istituti Clinici Scientifici Maugeri IRCCS, Milan, Italy; 2 Department of Clinical Sciences and Community Health, University of Milan, Milan, Italy

**Keywords:** ecological momentary assessment, hereditary angioedema, rare diseases, feasibility, acceptability

## Abstract

**Introduction:**

Hereditary angioedema (HAE) is a rare disease imposing a significant quality of life burden. Affect monitoring via Ecological Momentary Assessment (EMA) could offer personalized psychological support by collecting repeated, ecological data in real-life, overcoming the limitations of traditional methods. This study assessed the feasibility and acceptability of an EMA protocol for affect monitoring in HAE patients vs. healthy controls (CTR).

**Methods:**

HAE patients and CTR were recruited for a 16-week EMA study. Participants received weekly EMA surveys assessing affect via REDCap™. Feasibility was evaluated through recruitment, response, and completion rates. Acceptability was assessed via a post-study questionnaire through a visual analogue scale ranging from 1 to 100.

**Results:**

Twenty-eight Caucasian subjects were contacted, 12 HAE [median age: 50 (22) years, 5 males] and 14 CTR [age: 30 (32) years, 6 males] agreed to participate, resulting in a recruitment rate of 93%. Response and completion rates were ≥92% and ≥96% respectively in both groups. Completion time was brief and did not differ between groups [HAE: 1′ 28″ (29″) vs. CTR: 1′ 15′ (15″), *P* = 0.274]. The protocol was considered acceptable by both groups [HAE: rate 83.5 (18.8) vs. CTR: 72.0 (13.0), *p* = 0.27] with HAE rating the experience as helpful [79 (39.8)] and thought-provoking [67 (33)].

**Conclusion:**

EMA is a highly feasible and acceptable method for affect monitoring in HAE. The presence of a rare disease does not appear to be a barrier to its application, supporting its use in this clinical setting.

## 1 Introduction

Hereditary Angioedema (HAE) is a rare genetic disease resulting from deficiencies (type 1) or dysfunctions (type 2) in the C1 inhibitor protein (1). HAE causes recurrent and unpredictable swelling episodes (i.e., angioedema attacks) that may affect limbs, face, airways, and the intestinal tract (2). In the most severe instances, attacks affecting the airways can be life-threatening, as swelling may lead to asphyxiation and death (3). Currently, Long-Term Prophylactic (LTP) treatments and On-Demand Therapy (ODT) are available for symptoms management.

Beyond the physical components, HAE may have significant psychological consequences, including stress, anxious and depressive symptoms, due to the unpredictable and recurrent nature of attacks, fear of fatal consequences, and the difficulties in managing physical symptoms (4). Many patients also report stress and anxiety as triggers of HAE attacks (5), suggesting a possible bidirectional relationship between HAE and psychological symptoms (6).

To date, the only attempts to investigate the relationship between HAE, psychological variables and patients' quality of life (QoL) have been based on Patient Reported Outcomes Measures (PROMs) (7–9). However, as any other retrospective self-reported instrument, PROMs often lead to recall bias (10) that can significantly affect the validity and reliability of the collected data. To overcome this limitation, the Ecological Momentary Assessment (EMA) method has been chosen (11). EMAs collect data on people's behaviors, experiences and psychological states in their natural environment through emails, alongside other methods like text messaging or dedicated mobile apps. It overcomes the limitations of traditional questionnaires, minimizing recall bias and providing a more accurate understanding of daily experiences by administering short surveys via electronic devices at specific scheduled times to improve ecological validity (11).

This method also offers a better insight into the dynamic ebb and flow of people's experiences, that cannot be captured with traditional questionnaires in cross-sectional or pre-post studies (12).

The EMA method has been demonstrated to be feasible in the context of non-organic and organic diseases, such as in studies examining patients with psychotic-spectrum disorders (13), suicide risk (14), and the temporal dynamics between cortisol and depression (15), evaluating the associations between daily physical activity and symptoms in breast cancer patients and sub-arachnoid hemorrhage (16, 17). Despite the increasing number of studies based on this approach, to date EMA has never been tested in patients affected by rare diseases.

Starting from the supposed link between HAE attacks and psychological aspects, collecting repeated information about the patient's emotional activations in real-life contexts could be beneficial for a better understanding of how the disease can impact emotional well-being and vice versa.

### 1.1 Purpose of the study

The primary aim of this study was to assess the feasibility and acceptability of collecting longitudinal EMA-based data on emotional states in patients with HAE. Given that the substantial burden associated with HAE, including unpredictable physical attacks and psychological distress, could plausibly act as a barrier to this methodology, a healthy control (CTR) group was included for comparison.

## 2 Methods

### 2.1 Participants and study design

A two-arm, parallel-group, observational controlled study was conducted between September 2023 and January 2024. All participants started and finished the study at the same time.

A sample of patients with a confirmed diagnosis of C1 inhibitor deficiency HAE Type I or Type II (1) who belonged to the Italian Network for Hereditary and Acquired Angioedema (ITACA)^1^ referring for clinical treatments to a tertiary care center, were consecutively enrolled during routine clinical visits. The CTR group was recruited among workers of the hospital and patients' relatives.

Inclusion criteria for both groups were: (1) being 18 years old or older; (2) speaking and understanding Italian; (3) possessing a personal electronic device (smartphone, tablet, computer) set to receive push notifications. Exclusion criteria were: (1) reported presence of cognitive impairments; (2) diagnosis of mental diseases; (3) inability to provide written informed consent.

The study was approved by the relevant Ethics Committee and all participants provided a written informed consent for the study procedures at enrollment.

This study followed the guidelines of the Checklist for Reporting Results of Internet E-Surveys (CHERRIES) (18) (see Supplementary File S1).

Participants did not receive any kind of compensation for participating in this study.

### 2.2 Sociodemographic and clinical measures

All participants completed a socio-demographic questionnaire at enrollment. For the patient group, clinicians collected HAE diagnosis and actual use of LTP treatments from hospital medical records. Patients were also required to complete at baseline the Angioedema Control Test (AECT) (19) and Angioedema Quality of Life (AE-QoL) (20) questionnaires.

The AECT is a 4-item questionnaire designed to assess symptom control in patients with recurrent angioedema attacks, while the AE-QoL consists of 17 items to measure health-related quality of life impairment in these patients. Patients were asked to record HAE attacks in the ITACA registry (https://www.ClinicalTrials.gov ID NCT03828279), the Italian prospective registry for angioedema patients. The registry allows patients to note the occurrence, duration, gravity, and other details regarding the attacks.

### 2.3 Ecological momentary assessment

The EMA method involved the collection of short questionnaires from participants via email using the Research Electronic Data Capture (REDCap™) (21) platform, a web application designed for data collection and management. The decision to collect data via email was made due to institutional concerns about the security and privacy of the data collected via third-party smartphone applications. To respect these requirements, we opted to use REDCap, an institutional software already used in previous EMA studies, that would guarantee the highest level of confidentiality, that only allows data collection on email. While email might not be the most common method for EMA due to potential delays and lower response rates compared to apps or text messages, it can still be a viable option (22).

The study procedures and surveys were pre-tested and approved by the research team. At enrollment, participants attended a training session to familiarize with the EMA method and provided their email address to receive the surveys. Push notifications were enabled to ensure timely responses.

Both HAE patients and CTR responded to the EMAs once a week for 16 consecutive weeks, within 90 min from receipt. This timeframe allowed for postponement due to personal or work commitments. If participants did not respond within this time, reminder emails were automatically sent every 90 min, up to three times per survey. Participants who did not complete the weekly survey on time would receive no further reminders and their participation in the study would be terminated.

**Figure 1:**
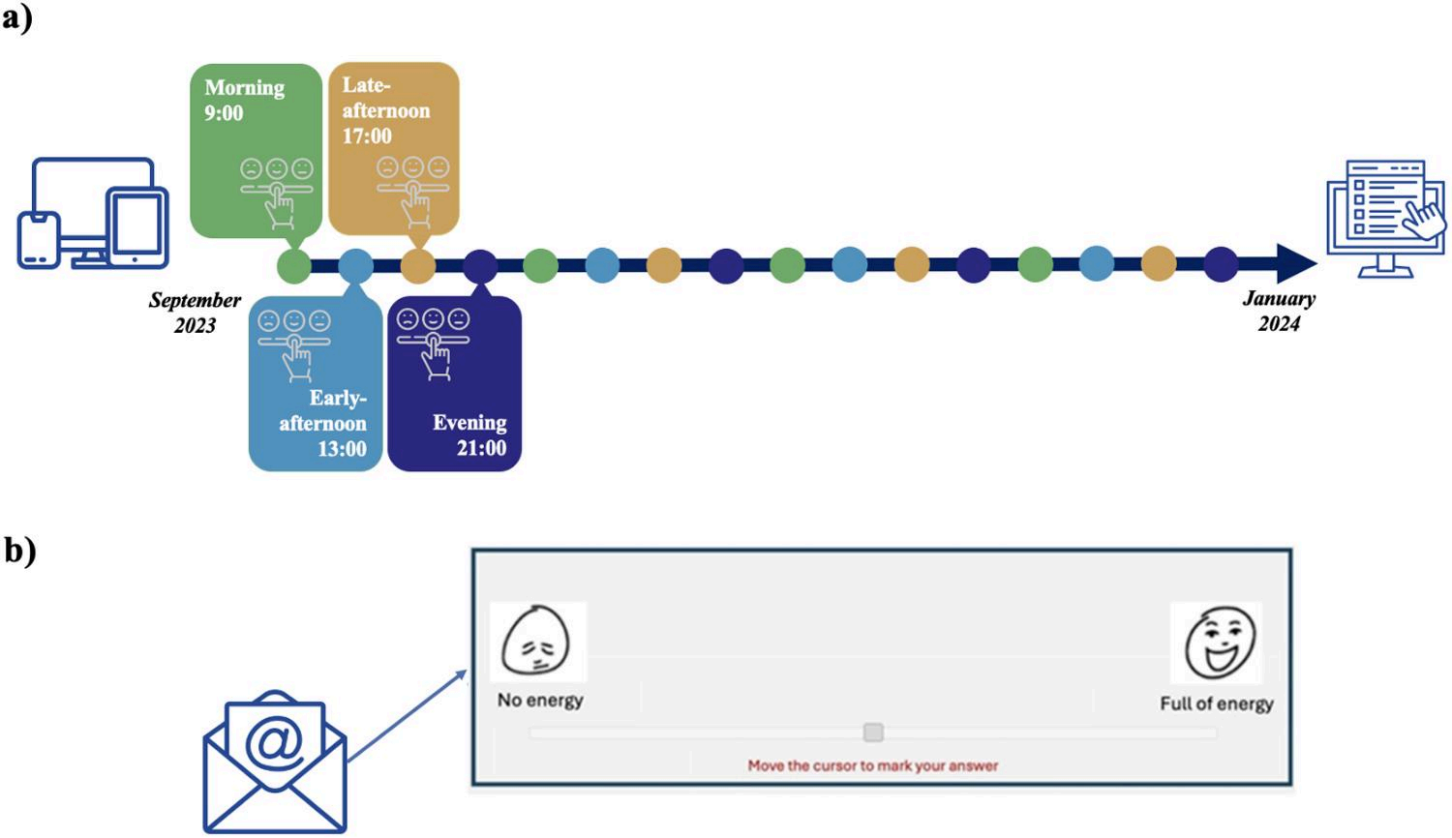
Study timeline. Graphical representation of the study timeline **(a)** and example of an item included in the weekly surveys **(b)**. Icons from: “electronic devices icon” by xnimrodx, “unhappy to happy scale icon” by Freepik, “online survey icon” by Maan Icons and “email icon” by Smashicons, licensed under Flaticon License.

Duplicate survey entries were avoided by relying on a pseudonymized user ID and date of survey submission, and by not allowing surveys to be reopened after submission. Over the 16 weeks of the study, the EMA distribution schedule was varied at different days and times to maximize data reliability by avoiding response bias associated with specific times or days of the week (Figure 1a) (10).

The weekly surveys were based on the Positive Activation, Negative Activation and Valence Short Scale (PANAVA-KS) (23), with the adjunct of validated emoticons (24), specifically developed for the ecological assessments. This validated scale assesses two general activation systems of affect (PA/NA) and the classical valence dimension (VA) (25) known to fluctuate during the subjects' daily experiences. It includes 10 different items, each represented by a pair of emoticons positioned on the edges of a slider, depicting bipolar adjectives (e.g., Stressed-Relaxed, Enthusiastic-Bored, etc.) (see Supplementary File S2). The items were presented on a single page, the order of presentation was not randomized, and the surveys did not use adaptive questioning.

Every time participants received the weekly prompt, they were asked to indicate their current emotional state by positioning the slider cursor on a continuum between the two opposite emotions (Figure 1b). The presence of the emoticons facilitates an intuitive understanding and reporting of the participants' emotional states and leverages the emotive expressiveness of emoticons to capture subtle variations in emotional activation and valence (26). Participants were forced to answer any of the 10 sliders and were allowed to change their responses until the survey submission. A completeness check before the submission was automatically provided by the system to avoid missing data and the submission of incomplete answers.

### 2.4 Feasibility measures

The feasibility of the EMA protocol was evaluated in terms of recruitment, response, completion, and retention rates [%].

The recruitment rate is the ratio between individuals who agreed to participate and the eligible recruited ones. This measure provides insights into the study's accessibility and appeal (27).

The response rate represents the percentage of individuals who completed the surveys in relation to the total number of participants (28). It is used to assess the effectiveness of the data collection method and the participants' overall engagement (28). Though there is no specific threshold or standard for defining a high response rate, a rate of 80% or higher is considered excellent (28).

The completion rate refers to the proportion of respondents who completed the survey out of the total number of respondents who entered it (29). This metric helps to evaluate participants' engagement, cooperation, and survey-related issues.

Finally, the retention rate is the percentage of participants who remained in the study from the beginning to the end, completing all 16 surveys and it represents the ability of the study to maintain a cohort of participants over time (30). However, due to the study design, retention and response rates at the end of the study are the same.

The time to respond to each EMA survey, the number of reminders, as well as the survey return times (i.e., duration between survey deployment and its completion) along the 16 study weeks were also recorded as other relevant feasibility indicators. The survey return time and the time to respond to each EMA were compared across the four different deployment times (i.e., morning, early afternoon, late afternoon, and evening).

### 2.5 Acceptability measures

The acceptability was measured by administering a 7-item questionnaire at the end of the study to all participants (31). Each item evaluated a different aspect of the subject's experience (i.e., “Positive”, “Negative”, “Time-consuming”, “Draining”, “Helpful”, “Thought-provoking” or “Annoying/Frustrating”) scored on a visual analogue scale ranging from 1 to 100 (1 = strongly disagree; 100 = strongly agree). No recognized threshold to define a good acceptability is available for the adopted questionnaire.

Participants were also asked to rate their interest in participating in future similar studies using a 7-point Likert scale ranging from 0 (completely disagree) to 6 (completely agree). Then, they were asked about their preferred frequency for survey distribution through a multiple-choice question (adequate; more frequently; less frequently). The presence of technical issues was also investigated.

Lastly, participants were asked to provide their feedback through two open-ended questions, one about what they liked and disliked, and the second one about any suggestions for future similar studies.

### 2.6 Data analysis

Descriptive statistics were used to assess participants' characteristics, feasibility and acceptability indicators. Categorical variables were summarized as absolute numbers and percentages. Continuous variables were given as median [interquartile range] values. The Mann–Whitney *U*-test and Chi-squared test were conducted to evaluate differences between HAE and CTR according to the type of variables. Friedman test was used to examine possible within-group differences between sessions in the response and return time.

The two-sided *P*-value <0.05 determined the statistical significance. Responses to open-ended interview questions were summarized and reviewed multiple times by two study authors to identify patterns among responses.

## 3 Results

### 3.1 Recruitment and characteristics of the participants

**Figure 2:**
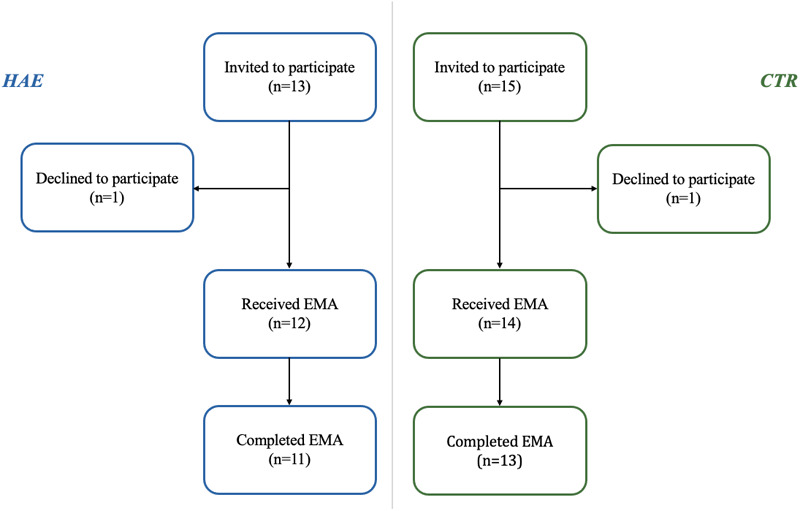
Study flowchart. Study flowchart related to the application of EMA surveys in patients with HAE and healthy individuals.

A total of 28 individuals were invited to participate, including 13 patients with HAE and 15 CTR. The recruitment of all participants was completed in less than one month. Twenty-six out of the 28 contacted individuals agreed to participate, resulting in a recruitment rate of 93%. The flowchart of the study is shown in Figure 2.

No significant differences were found in age, sex, and educational level between HAE patients and CTR (Table 1).

**Table 1:** Characteristics of the study populations.

Characteristics	HAE (*n* = 12)	CTR (*n* = 14)	*p*-value
Age (years)	50 [22]	30 [32]	0.27
Sex (male/female)	5/7	6/8	1.00
Education level (years)	15 [5]	18 [3]	0.08
AECT (0–16)	15.0 [2.3]	–	–
AE-QoL (17–85)	28.0 [13.5]	–	–
Diagnosis (type 1/type 2)	10/2	–	–
LTP (yes/no)	8/4	–	–
HAE attacks (yes/no)	7/5	–	–

Continuous data are presented as median [IQR], categorical data are presented as absolute numbers. HAE, hereditary angioedema; CTR, healthy controls; AECT, angioedema control test; AE-QoL, angioedema quality of life; LTP, long term prophylaxis.

Participants in the HAE group showed good disease control, with a median AECT score of 15 [2.3], and a relatively low impairment in QoL, as indicated by the median AE-QoL score of 28 [13.5]. The 67% of patients were treated with LTP. During the entire study period, 58% of HAE patients experienced at least one attack (Table 1).

Most of the participants in the HAE group (75%) and in the CTR group (93%) reported using e-mail daily. None of the participants has entered an EMA study before.

### 3.2 Feasibility

A total of 400 records were received from the 26 participants. In the HAE group, the response rate was 100% until the 6th week and decreased to 92% from the 7th week to the end of the study, whereas in the CTR group, the response rate ranged from 100% during the first 12 weeks, and decreased to 93% in the last 4 weeks. So, 92.3% of the participants completed the entire study protocol determining an overall retention rate of 92% (only two participants dropped out).

In the HAE group, the completion rate was 100% for the first seven weeks, dropped to 92% in the 7th week, but bounced back to 100% from the 8th week until the end of the study. In the CTR group, the completion rate was 100% for the first 13 weeks, dropped to 93% in the 13th week, but returned to 100% for the remaining period. One person dropped out in each group, resulting in an average completion rate of 99.7% by the end of the study. In particular, the HAE patient dropped out after six weeks due to technical issues related to the automated sending system, while the healthy subject withdrew after twelve weeks due to a personal loss of motivation.

Overall, the two groups were not different in terms of time required to complete the EMAs. The median amount of time patients took to complete each survey (1′ 28″ [0′ 29″]) was not different to the one taken by healthy individuals [1′ 15′ (0′ 15″), *P* = 0.274]. No differences were found during the 16 weeks in the time to complete the surveys in the patients' group (*P* = 0.065) and CTR (*P* = 0.136). Among the 400 received, only one survey per group was submitted in less than 20 s. Around 96% of all surveys were completed in 3 min or less. Participants generally took more time to complete the first EMA [2′ 30″ (1′ 57″) for patients and 2′ 00″ (1′ 00″) for healthy individuals] compared to the following ones. There was only a significant reduction in the time to complete the survey between the first and the second session in the CTR group [2′ 00″ (1′ 00″) vs. 1′ 00″ (0′ 53″), *P* = 0.010], but not in the HAE group [2′ 30″ (1′ 57″) vs. 2′ 00″ (1′ 00″), *P* = 0.477].

The four different time slots (i.e., morning, early afternoon, late afternoon, and evening) did not show differences in response times between the HAE (*P* = 0.425) and the CTR group (*P* = 0.787).

**Figure 3:**
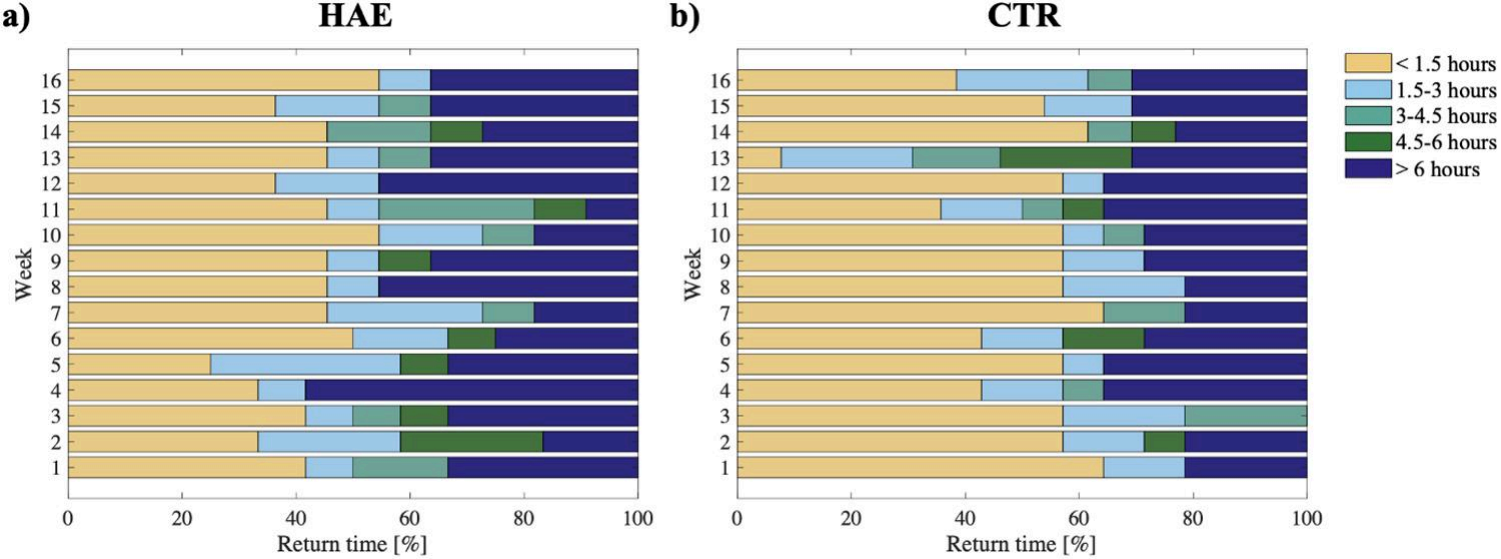
Survey's return time. Return time of EMA surveys for each week in the group of HAE patients **(a)** and in the group of healthy individuals **(b)**.

During the 16 weeks, the median percentage of the participants who responded to the surveys within 1.5 h from the received prompt (no need for reminders) was 42 [8] % for the HAE group and 57 [14] % for the CTR group. Thirty-three percent [10] of HAE subjects and 29 [9] % of CTR responded after 6 h from the deployment of the surveys. These percentage values were stable during the study (Figure 3). A delay in the return time was observed only in the 13th week among the CTR.

The return time was not influenced by the deployment time in both groups.

### 3.3 Acceptability

Twenty-five (96%) participants completed the acceptability questionnaire at the end of the study to evaluate their experience with the EMA. Overall, in the 7-item acceptability questionnaire, all the participants rated the experience as acceptable, but the HAE group reported significantly higher values compared to the CTR group in the “Thought-provoking” item (Table 2).

**Table 2:** Acceptability findings and comparisons between HAE patients and healthy individuals.

Acceptability ratings	HAE (*n* = 12)	CTR (*n* = 13)	*p*-value
1 – Positive experience (0–100)	83.5 [18.8]	72.0 [13.0]	0.270
2 – Negative experience (0–100)	7.0 [14.8]	13.0 [22.0]	0.270
3 – Time-consuming (0–100)	9.0 [10.8]	23.0 [40.0]	0.060
4 – Draining (0–100)	4.5 [11.8]	13.0 [34.0]	0.225
5 – Helpful (0–100)	79.0 [39.8]	50.0 [37.0]	0.068
6 – Thought-provoking (0–100)	67.0 [33.0]	50.0 [23.0]	0.046
7 – Annoying/frustrating (0–100)	10.0 [14.5]	25.0 [39.0]	0.186

Data are presented as median [IQR].

The post-surveys feedback questionnaire included a question about the participants' interest in participating in future similar studies, where all HAE patients expressed a positive response, while 25% of the CTR expressed a neutral or negative feedback.

Regarding the survey distribution frequency, most of the participants (88%) stated that one survey per week was adequate with no significant differences between the two groups. Only 3 participants experienced technical issues related to the delivery of the surveys. Two complained that the email always ended up in the spam folder, while the third said he never received any email.

Only five participants provided answers to the open-ended questions regarding their preferences, the changes they would make to improve the data collection, and their overall suggestions. Two participants appreciated that the study required them to focus on their emotional states and allowed them to compare different time points to observe changes. Three of them disliked the fact that some questions were repetitive and not specific enough, and criticized the decision to use emails rather than a smartphone app to receive EMAs.

## 4 Discussion

To the best of our knowledge, this is the first study testing the EMA approach on patients with a rare disease, as HAE.

Two-thirds of the patients in the study were on prophylactic therapy, meaning that their condition was severe enough to require LTP (32), which, although it does not guarantee the complete absence of attacks, significantly improves disease management and reduces their frequency and severity. This results in a relatively low impairment in quality of life as indicated by the AECT and AE-QoL scores.

In terms of feasibility of the EMA approach, the high recruitment, retention, response, and completion rates, together with the short survey return time and the low need for reminders achieved among patients and healthy participants demonstrate the suitability of the proposed method. The recruitment process was completed in less than a month, suggesting that the study design was effective in attracting and enrolling participants. Moreover, only two subjects withdrew from the study after the training session, indicating that there were minimal barriers to the initial engagement. The recruitment rate of 93% indicates the high willingness and motivation of the two samples to participate in the study. This may have been fostered by the fact that subjects were invited to participate to a pre-study session where the researchers explained them the aims of the research enhancing their engagement and interest (33). For patients only, they may have been interested in participating because of their personal relevance to the advancement of knowledge about their illness (34), which is particularly strong for those affected by a rare disease.

The retention rate of around 92% in both groups indicates that participants were generally compliant with the four-month study protocol, the weekly EMA, the number of reminders, and the delivery schedule, suggesting a low impact of the research design on everyday life (35). Notably, this retention rate not only was higher than those obtained in other studies based on EMA techniques (36, 37), but was reached despite the lack of incentives, that are frequently used to support participants' engagement (38). Possible explanations for this achievement may be found in the quite long intervals between the assessments (one week from one to the other) (39); the small number of items per evaluation (i.e., 10 items); the very short time required to complete the weekly surveys (i.e., less than 3 min) (40, 41); and the type of items (i.e., emoticons that do not require semantic analysis to be understood).

The response rate was higher than 92% for the entire duration of the study in both groups, suggesting a good participant engagement and the consequent validity of the collected data. Such rate is higher than the one observed in similar studies based on the use of smartphone apps (42, 43), which are the most common used method for the EMA studies. This difference may be due to the participants’ familiarity with the use of email compared to the use of a never-before-used app specifically dedicated to the study. These data agree with those obtained by previous studies using emails to collect electronic PROMs (31, 43). Moreover, the possibility of accessing the surveys from the preferred personal devices, and to switch between them at any time during the study may have also favored the response rate.

Return times were consistent throughout the study, except for week 13, when most controls responded with a delay (from 1.5 h to more than 6 h). It is worth noting that the 13th assessment coincided with an Italian public holiday (1 January), so some delay was expected. However, the return time for HAE patients did not differ from the previous or subsequent assessments. A possible reason for this group difference could be the level of commitment and motivation. HAE participants may have been more motivated due to the personal relevance of the study, leading them to follow the instructions given during the pre-study meeting more carefully and to respond as quickly as possible.

At the end of the study, all participants were asked to complete a feedback survey focusing on the perceived acceptability of the EMA method. Similar to what Rogers and colleagues (31) found in another clinical sample, the EMA approach was rated as a positive experience by both groups: the assessment was not perceived as time-consuming, tiring or annoying/frustrating. Good ratings were also given for the helpfulness and thought-provoking nature of the study. However, a significant difference was found between patients and healthy volunteers on the “Thought-provoking” item, suggesting that patients were more likely to focus on their emotional states. In addition, they felt more engaged with the surveys as they facilitated their awareness of real-life changes in affect over time. In addition, most participants reported to be available to participate in future long-term monitoring using EMAs and affirmed that the once-a-week frequency is suitable.

Finally, very few participants reported technical issues, such as prompts sent to the spam folder or not received at all. For these reasons, they suggested changing the survey distribution platform from emails to other instant messaging applications.

The results of the present study have several implications for future clinical and research practice in HAE population. Firstly, EMA could be used in clinical trials to evaluate the effectiveness of new therapies in reducing patient's disease burden in real world. Secondly, future studies based on the use of dedicated apps, will allow to collect more fine-grained data to investigate the temporal relationship between psychological states, daily events and the HAE attacks to better explain the link between triggers and symptoms.

### 4.1 Strengths and limitations

For this pilot study, the prompts were sent via email instead of through a dedicated application. This approach offered advantages to both participants and researchers. Participants could respond using their preferred electronic devices and switch between them as needed. Researchers benefited from using REDCap™, as this avoided costs, maintenance issues, software interoperability problems, and data security concerns (22). However, REDCap™ also has limitations, such as significant staff time required for setup and monitoring, and emails ending up in spam folders or not being received. Another limitation was imposed by the functionality of REDCap's Automated Survey Invitations. In our longitudinal design, delivery of each survey depended on completion of the preceding one. Consequently, if a participant failed to complete a single weekly survey, the automated invitation sequence for that individual was terminated, meaning they would not receive any further surveys for the remainder of the study. A further limitation is that the assessment of acceptability was conducted only once, at the end of the study period. Consequently, we were unable to formally assess the longitudinal change in patient perception and potential respondent fatigue. Finally, we opted for a lower frequency of assessments than is usual for EMA practice, but over a longer period of time. This was mainly an attempt to avoid overburdening patients in their daily lives.

From the physician standpoint, it is unrealistic to expect busy clinicians to manually review weekly data returns for all patients. However, it is important to point out that the physician would not be required to evaluate survey results in real time; rather, data collection would more accurately reflect the events occurring in the patient's daily life. This pilot study provides preliminary evidence supporting the feasibility of this approach, suggesting that assessing a more multidimensional disease burden may be less complex than commonly assumed.

## 5 Conclusions

The EMA approach is quite new in the worldwide research and clinical landscape, so the definition of guidelines to collect reliable data is mandatory. To our knowledge, the present study is the first one to assess the feasibility and acceptability of this approach in HAE patients compared to healthy subjects, suggesting that the presence of a rare and chronic illness does not interfere with a repeated, longitudinal assessment in real life.

These findings encourage and support the future use of EMA for the long-term monitoring of symptoms and emotional states in order to identify patterns of fluctuations related to symptoms onset, treatment effectiveness, and overall disease management in HAE patients, and other diseases. Such an approach could also help to identify unmet needs and promote personalized psychological support to provide a better multidisciplinary care.

## Data Availability

The datasets presented in this study can be found in online repositories. The names of the repository/repositories and accession number(s) can be found below: https://doi.org/10.6084/m9.figshare.29880173.

## Footnotes

^1^Italian Network for Hereditary and Acquired Angioedema - ITACA. Available online at: https://angioedemaitaca.org/

## References

[1] Maurer M Magerl M Betschel S Aberer W Ansotegui IJ Aygören-Pürsün E The international WAO/EAACI guideline for the management of hereditary angioedema—the 2021 revision and update. World Allergy Organ J. (2022) 15:100627. 10.1016/j.waojou.2022.100627 35497649 PMC9023902

[2] Bork K Meng G Staubach P Hardt J. Hereditary angioedema: new findings concerning symptoms, affected organs, and course. Am J Med. (2006) 119:267–74. 10.1016/j.amjmed.2005.09.064 16490473

[3] Santacroce R D’Andrea G Maffione AB Margaglione M d’Apolito M. The genetics of hereditary angioedema: a review. J Clin Med. (2021) 10:2023. 10.3390/jcm10092023 34065094 PMC8125999

[4] Eyice Karabacak D Demir S Yeğit OO Can A Terzioğlu K Ünal D Impact of anxiety, stress and depression related to COVID-19 pandemic on the course of hereditary angioedema with C1-inhibitor deficiency. Allergy. (2021) 76:2535–43. 10.1111/all.14796 33650198 PMC8014132

[5] Craig T. Triggers and short-term prophylaxis in patients with hereditary angioedema. Allergy Asthma Proc. (2020) 41:S30–4. 10.2500/aap.2020.41.200058 33109323

[6] Busse PJ Christiansen SC. Hereditary angioedema. N Engl J Med. (2020) 382:1136–48. 10.1056/NEJMra1808012 32187470

[7] Hews-Girard J Goodyear MD. Psychosocial burden of type 1 and 2 hereditary angioedema: a single-center Canadian cohort study. Allergy Asthma Clin Immunol. (2021) 17:61. 10.1186/s13223-021-00563-0 34187550 PMC8244202

[8] Mendivil J Murphy R de la Cruz M Janssen E Boysen HB Jain G Clinical characteristics and burden of illness in patients with hereditary angioedema: findings from a multinational patient survey. Orphanet J Rare Dis. (2021) 16:94. 10.1186/s13023-021-01717-4 33602292 PMC7893968

[9] Black N Jenkinson C. Measuring patients’ experiences and outcomes. Br Med J. (2009) 339:b2495. 10.1136/bmj.b2495 19574317

[10] Jager KJ Tripepi G Chesnaye NC Dekker FW Zoccali C Stel VS. Where to look for the most frequent biases? Nephrology. (2020) 25:435–41. 10.1111/nep.13706 32133725 PMC7318122

[11] Shiffman S Stone AA Hufford MR. Ecological momentary assessment. Annu Rev Clin Psychol. (2008) 4:1–32. 10.1146/annurev.clinpsy.3.022806.091415 18509902

[12] Schneider S Stone AA. Ambulatory and diary methods can facilitate the measurement of patient-reported outcomes. Qual Life Res. (2016) 25:497–506. 10.1007/s11136-015-1054-z 26101141 PMC4689672

[13] Moitra E Gaudiano BA Davis CH Ben-Zeev D. Feasibility and acceptability of post-hospitalization ecological momentary assessment in patients with psychotic-spectrum disorders. Compr Psychiatry. (2017) 74:204–13. 10.1016/j.comppsych.2017.01.018 28231480 PMC5369417

[14] Morgiève M Genty C Azé J Dubois J Leboyer M Vaiva G A digital companion, the emma app, for ecological momentary assessment and prevention of suicide: quantitative case series study. JMIR Mhealth Uhealth. (2020) 8:e15741. 10.2196/15741 33034567 PMC7584985

[15] Booij SH Bos EH de Jonge P Oldehinkel AJ. The temporal dynamics of cortisol and affective states in depressed and non-depressed individuals. Psychoneuroendocrinology. (2016) 69:16–25. 10.1016/j.psyneuen.2016.03.012 27017429

[16] Whitaker M Welch WA Fanning J Santa-Maria CA Auster-Gussman LA Solk P Using ecological momentary assessment to understand associations between daily physical activity and symptoms in breast cancer patients undergoing chemotherapy. Support Care Cancer. (2022) 30:6613–22. 10.1007/s00520-022-07071-w 35488902 PMC11934233

[17] de Vries EA Heijenbrok-Kal MH van Kooten F Giurgiu M Ribbers GM van den Berg-Emons RJG Unraveling the interplay between daily life fatigue and physical activity after subarachnoid hemorrhage: an ecological momentary assessment and accelerometry study. J Neuroeng Rehabil. (2023) 20:127. 10.1186/s12984-023-01241-5 37752550 PMC10521384

[18] Eysenbach G. Improving the quality of web surveys: the checklist for reporting results of internet E-surveys (CHERRIES). J Med Internet Res. (2004) 6:e34. 10.2196/jmir.6.3.e34 15471760 PMC1550605

[19] Weller K Donoso T Magerl M Aygören-Pürsün E Staubach P Martinez-Saguer I Validation of the angioedema control test (AECT)—a patient-reported outcome instrument for assessing angioedema control. J Allergy Clin Immunol Pract. (2020) 8:2050–7.e4. 10.1016/j.jaip.2020.02.038 32173507

[20] Weller K Groffik A Magerl M Tohme N Martus P Krause K Development and construct validation of the angioedema quality of life questionnaire. Allergy. (2012) 67:1289–98. 10.1111/all.12007 22913638

[21] Harris PA Taylor R Thielke R Payne J Gonzalez N Conde JG. Research electronic data capture (REDCap)—a metadata-driven methodology and workflow process for providing translational research informatics support. J Biomed Inform. (2009) 42:377–81. 10.1016/j.jbi.2008.08.010 18929686 PMC2700030

[22] Dahr YE Perquier F Moloney M Woo G Dobrin-De Grace R Carvalho D Feasibility of using research electronic data capture (REDCap) to collect daily experiences of parent-child dyads: ecological momentary assessment study. JMIR Form Res. (2023) 7:e42916. 10.2196/42916 37943593 PMC10667976

[23] Nr. “Qualität des Erlebens in Arbeit und Freizeit” Untersuchungen mit der Experience Sampling Method Kurzskalen zur Erfassung der Positiven Aktivierung, Negativen Aktivierung und Valenz in Experience Sampling Studien (PANAVA-KS).

[24] Schreiber M Jenny GJ. Development and validation of the “lebender emoticon PANAVA” scale (LE-PANAVA) for digitally measuring positive and negative activation, and valence via emoticons. Pers Individ Dif. (2020) 160:109923. 10.1016/j.paid.2020.109923

[25] Russell JA. A circumplex model of affect. J Pers Soc Psychol. (1980) 39:1161–78. 10.1037/h0077714

[26] Kaye LK Malone SA Wall HJ. Emojis: insights, affordances, and possibilities for psychological science. Trends Cogn Sci. (2017) 21:66–8. 10.1016/j.tics.2016.10.007 28107838

[27] Blumenberg C Menezes AMB Gonçalves H Assunção MCF Wehrmeister FC Barros AJD. How different online recruitment methods impact on recruitment rates for the web-based coortesnaweb project: a randomised trial. BMC Med Res Methodol. (2019) 19:127. 10.1186/s12874-019-0767-z 31217008 PMC6585038

[28] Booker QS Austin JD Balasubramanian BA. Survey strategies to increase participant response rates in primary care research studies. Fam Pract. (2021) 38:699–702. 10.1093/fampra/cmab070 34213547

[29] Eysenbach G. Correction: improving the quality of web surveys: the checklist for reporting results of internet E-surveys (CHERRIES). J Med Internet Res. (2012) 14:e8. 10.2196/jmir.2042 PMC1550605 15471760

[30] Robinson KA Dinglas VD Sukrithan V Yalamanchilli R Mendez-Tellez PA Dennison-Himmelfarb C Updated systematic review identifies substantial number of retention strategies: using more strategies retains more study participants. J Clin Epidemiol. (2015) 68:1481–7. 10.1016/j.jclinepi.2015.04.013 26186981 PMC4658250

[31] Rogers ML. Feasibility and acceptability of ecological momentary assessment in a fully online study of community-based adults at high risk for suicide. Psychol Assess. (2021) 33:1215–25. 10.1037/pas0001054 34197164

[32] Craig T Busse P Gower RG Johnston DT Kashkin JM Li HH Long-term prophylaxis therapy in patients with hereditary angioedema with C1 inhibitor deficiency. Ann Allergy Asthma Immunol. (2018) 121:673–9. 10.1016/j.anai.2018.07.025 30056152

[33] David JN Rosa MP Santoro F Borges M. Considering context elements in pre-meeting systems. 2006 10th International Conference on Computer Supported Cooperative Work in Design. IEEE(2006). p. 1–6. 10.1109/CSCWD.2006.253211

[34] Trauth JM Musa D Siminoff L Jewell IK Ricci E. Public attitudes regarding willingness to participate in medical research studies. J Health Soc Policy. (2000) 12:23–43. 10.1300/J045v12n02_02 11184441

[35] Newington L Metcalfe A. Factors influencing recruitment to research: qualitative study of the experiences and perceptions of research teams. BMC Med Res Methodol. (2014) 14:10. 10.1186/1471-2288-14-10 24456229 PMC3903025

[36] Wang Y Jacques J Li Z Sibille K Cook R. Health outcomes among adults initiating medical marijuana for chronic pain: a 3-month prospective study incorporating ecological momentary assessment (EMA). Cannabis. (2021) 4:69–83. 10.26828/cannabis/2021.02.006 34671723 PMC8525881

[37] Porras-Segovia A Díaz-Oliván I Barrigón ML Moreno M Artés-Rodríguez A Pérez-Rodríguez MM Real-world feasibility and acceptability of real-time suicide risk monitoring via smartphones: a 6-month follow-up cohort. J Psychiatr Res. (2022) 149:145–54. 10.1016/j.jpsychires.2022.02.026 35276631

[38] Wrzus C Neubauer AB. Ecological momentary assessment: a meta-analysis on designs, samples, and compliance across research fields. Assessment. (2023) 30:825–46. 10.1177/10731911211067538 35016567 PMC9999286

[39] Mercieca-Bebber R Palmer MJ Brundage M Calvert M Stockler MR King MT. Design, implementation and reporting strategies to reduce the instance and impact of missing patient-reported outcome (PRO) data: a systematic review. BMJ Open. (2016) 6:e010938. 10.1136/bmjopen-2015-010938 27311907 PMC4916640

[40] Kato T Miura T. The impact of questionnaire length on the accuracy rate of online surveys. J Mark Anal. (2021) 9:83–98. 10.1057/s41270-021-00105-y

[41] Edney S Goh CM Chua XH Low A Chia J Koek DS Evaluating the effects of rewards and schedule length on response rates to ecological momentary assessment surveys: randomized controlled trials. J Med Internet Res. (2023) 25:e45764. 10.2196/45764 37856188 PMC10623229

[42] Howard AL Lamb M. Compliance trends in a 14-week ecological momentary assessment study of undergraduate alcohol drinkers. Assessment. (2024) 31:277–90. 10.1177/10731911231159937 36914966 PMC10822069

[43] Kinoshita S Hanashiro S Tsutsumi S Shiga K Kitazawa M Wada Y Assessment of stress and well-being of Japanese employees using wearable devices for sleep monitoring combined with ecological momentary assessment: pilot observational study. JMIR Form Res. (2024) 8:e49396. 10.2196/49396 38696237 PMC11099815

