# Supplementary material for: Ecological momentary assessments for patients with hereditary angioedema: a feasibility and acceptability controlled study
Source: Front Digit Health. 2026 Jan 12;7:1693550. doi: 10.3389/fdgth.2025.1693550 (PMC12833764; doi:10.3389/fdgth.2025.1693550)

### **Supplementary File 2.** EMA’s survey items for the Positive Activation, Negative Activation and Valence Short Scale (PANAVA-KS)


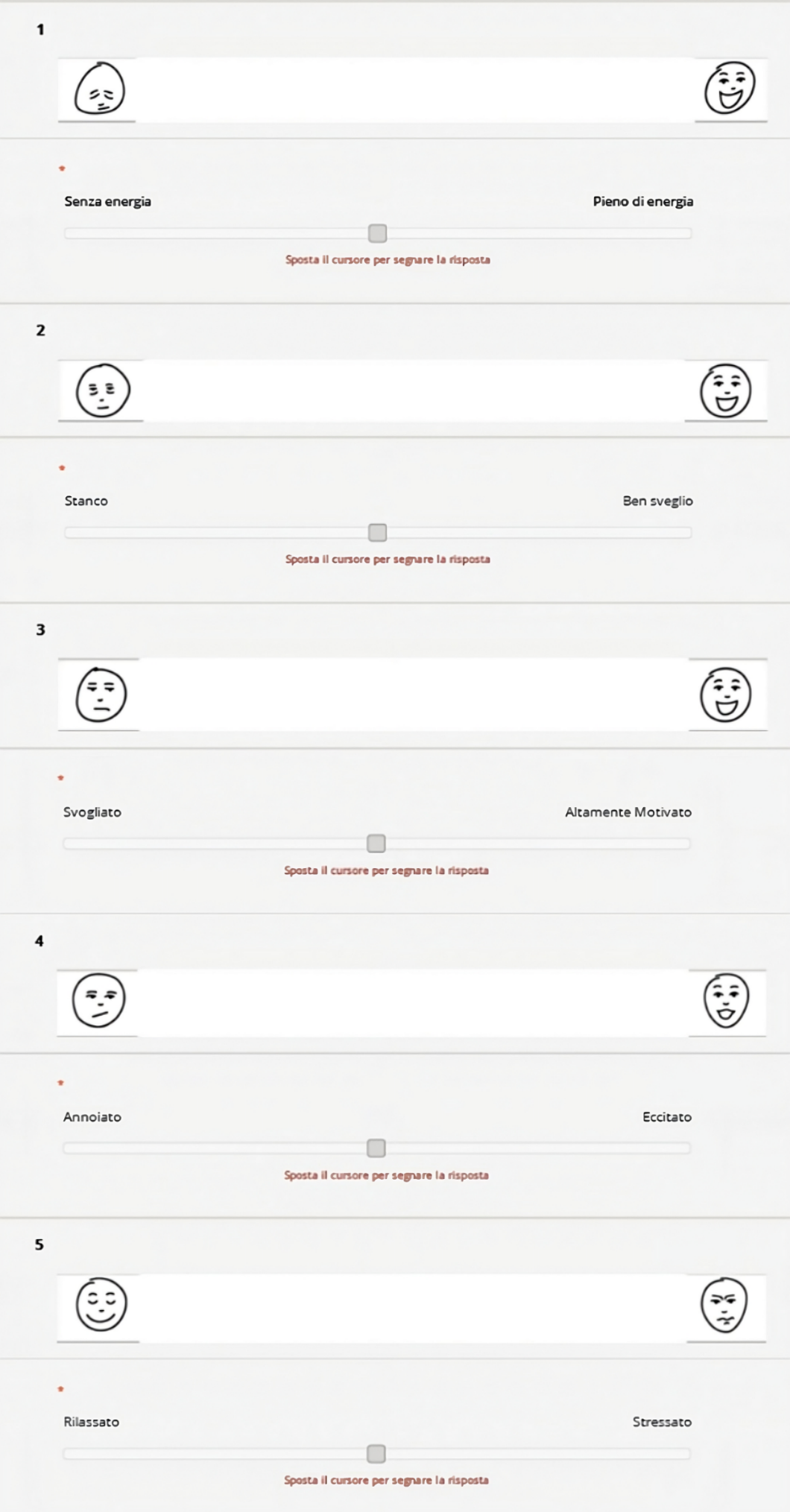


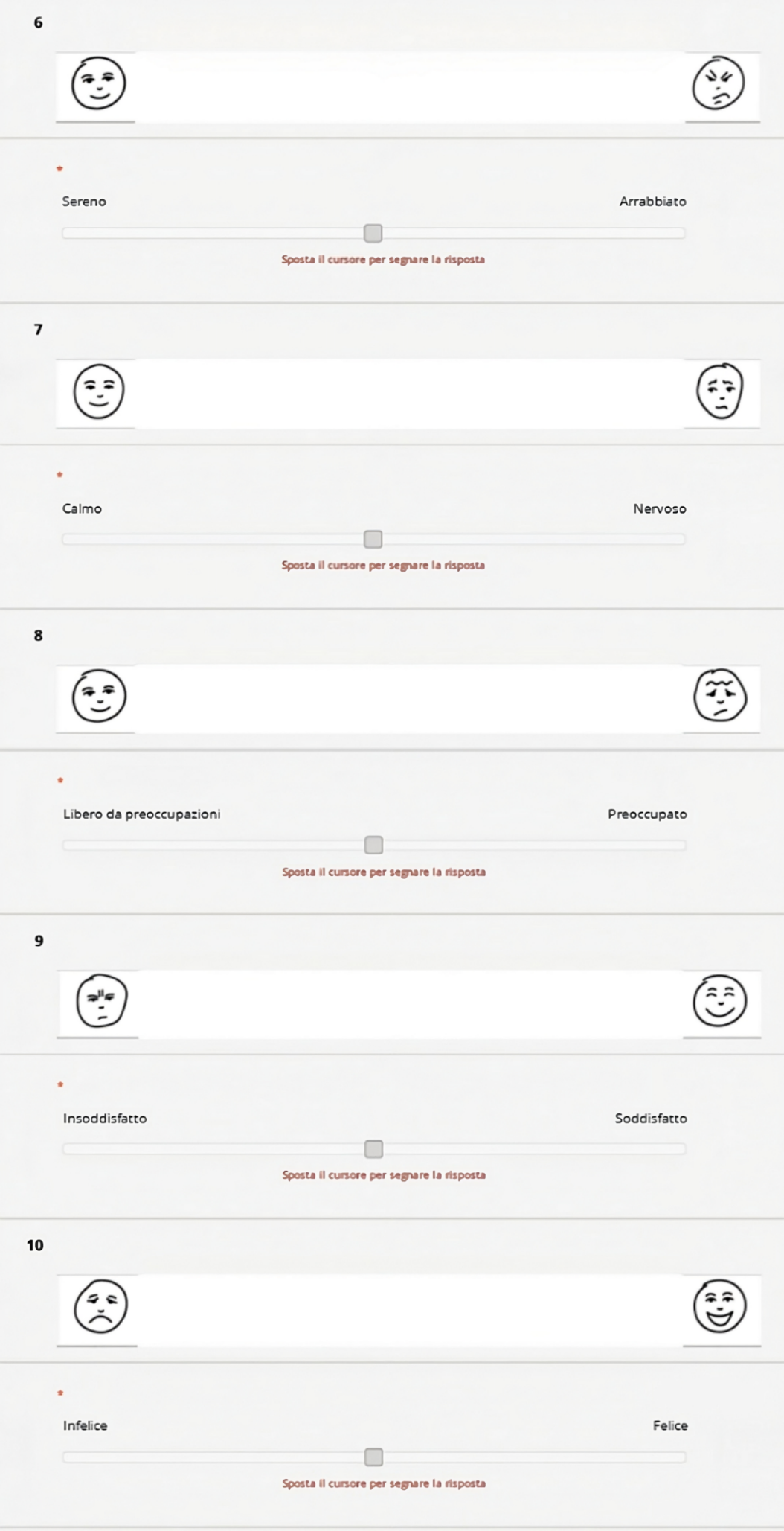

Supplement: Supplementary file 2 [file Supplementaryfile2.docx]
